# A practical method to target individuals for outbreak detection and control

**DOI:** 10.1186/1741-7015-11-36

**Published:** 2013-02-12

**Authors:** Gerardo Chowell, Cécile Viboud

**Affiliations:** 1 Mathematical and Computational Modeling Sciences Center, School of Human Evolution and Social Change, Arizona State University, Tempe, AZ, USA; 2 Division of International Epidemiology and Population Studies, Fogarty International Center, National Institutes of Health, Bethesda, MD, USA

**Keywords:** contact network, hotspot, dynamic network, contact pattern, wireless sensing devices, collocation ranking, class schedule, high school, influenza, disease transmission.

## Abstract

Identification of individuals or subpopulations that contribute the most to disease transmission is key to target surveillance and control efforts. In a recent study in *BMC Medicine*, Smieszek and Salathé introduced a novel method based on readily available information about spatial proximity in high schools, to help identify individuals at higher risk of infection and those more likely to be infected early in the outbreak. By combining simulation models for influenza transmission with high-resolution data on school contact patterns, the authors showed that their proximity method compares favorably to more sophisticated methods using detailed contact tracing information. The proximity method is simple and promising, but further research is warranted to confront this method against real influenza outbreak data, and to assess the generalizability of the approach to other important transmission units, such as work, households, and transportation systems.

See related research article here http://www.biomedcentral.com/1741-7015/11/35

## Background

The transmission potential of an infectious disease is directly related to the characteristics of the infectious agent, its host population and the local environment [1]. The contribution of these factors can be encapsulated in a single parameter that is key for disease control, namely, the 'reproduction number', which quantifies the average number of secondary cases generated by an infectious individual during the early epidemic phase [1]. Identification of individuals or subpopulations associated with high transmission potential is particularly useful to guide surveillance and control strategies, especially when resources are limited [2].

Understanding the complexity of dynamic human interactions and contact networks is crucial to identifying hotspots of disease transmission during an outbreak [3]. The dynamic social contact networks relevant for disease spread depends on a number of factors, including individual host characteristics (e.g, age, prior immunity, number of contacts), pathogen characteristics (transmission mode), characteristics of the space in which individuals interact (for example, confined versus open setting, room capacity), and the duration and proximity of human interactions.

Recent technological advances in miniature wireless sensing devices have allowed unobtrusive and unsupervised quantification of the dynamic network of human interactions in various settings, including schools [4-6], conferences [7], and hospitals [8]. In particular, these innovative technologies have increased our understanding of face-to-face contact patterns relevant for the spread of rapidly transmitted infectious agents [4,9]. Given the large amount of costly information captured by these devices, there is active debate on the minimum level of data that is required to capture the essence of disease transmission and to be sufficient to inform disease control [7,10].

## Performances of various indicators of social connectivity

A recent study by Smieszek and Salathé, published in *BMC Medicine *[11], used high-resolution contact-network data collected by wireless sensing devices during a 1-day period at a high school in the USA, combined with extensive epidemic simulations, to evaluate the effectiveness of several metrics to identify individuals who play a significant role in outbreak dissemination. The consolidated network dataset was limited to close proximity interactions, based on records indicating face-to-face contacts within a distance of less than 3 m at a certain point in time. The dataset also included location records indicating the presence of an individual in a specific classroom.

The authors then quantified the performances of a variety of indicators of social connectivity, which required different levels of information on the high-school contact network to identify individuals with high transmission potential. In particular, the authors introduced a low-cost indicator of social connectivity, based on the 'collocation-ranking method', which relies on the cumulative amount of time that an individual spends with other individuals in the same room, modulated by class size. Such information does not rely on the detailed structure of the high-school contact network, and can be retrieved from schedule data alone. The Smieszek and Salathé study relied on simulations of influenza transmission on the detailed high-school contact network to assess the performances of the different indicators, in terms of their ability to identify individuals at higher risk of infection and those with early disease onset.

## Findings and potential applications

Epidemic simulations showed that the simple schedule-based collocation ranking indicator clearly outperformed methods selecting individuals at random, and compared favorably with more data-hungry indicators. Because collecting reliable data about individual-level interactions is cumbersome and expensive to obtain at the community level, the authors proposed that their low-cost collocation method can be exploited for the design of sentinel surveillance systems, with the potential to quickly detect the onset of an infectious disease outbreak, and thereby optimize mitigation and prevention strategies. In particular, sentinel high-school students could be selected from those with high collocation ranking, and these could then be monitored for their infection status throughout the influenza season, and/or be prioritized for vaccination in the case of vaccine shortage, in an effort to stamp out an emerging outbreak.

## Limitations and future directions

This interesting proof-of-concept study by Smieszek and Salathé addressed social interactions within a high school, which is an important focus for seasonal and pandemic influenza transmission [12]. As acknowledged by the authors, a key limitation of this study is the lack of validation against epidemiological data from real school outbreaks. The simulation model used to evaluate the performances of the method is a conceptualized version of disease transmission, and although it is driven by real contact information, it remains one step removed from the actual disease-transmission process. A previous study combining outbreak data in an elementary school with contact-network information highlighted the importance of gender on influenza transmission, with children of the same gender infecting each other more frequently (reflecting assortative mixing) [4], an issue that was not considered by Smieszek and Salathé. Interestingly, school outbreak data have also shown that the exact location of children within the classroom does not matter, which supports the use of simple class-schedule information as proposed by Smieszek and Salathé [11] rather than the use of more detailed seating charts. Although there has been good progress overall in elucidating social interactions among school-age children, more studies are needed to address whether contact patterns, and hence transmission links, might differ between elementary and high schools.

Another limitation of the school-based study by Smieszek and Salathé [11] relates to the contribution of other units to disease transmission. About one-third of all influenza secondary-transmission events are believed to occur within households [13], whereas only 7 to 20% are thought to occur in schools [14]. Hence, estimating the relative infection risk of individuals in a variety of settings relevant for disease transmission, including schools, households, conferences, and transportation systems, will be important in future research. It is not clear how the method proposed by Smieszek and Salathé [11] could be generalized to household and work environments, where systematic 'schedules' are more difficult to obtain.

As noted by the authors, the transmission mode of influenza and other respiratory pathogens is not clearly understood, but probably involves a combination of direct contact and transmission by fomites and aerosols, which makes it difficult to capture the social network relevant for disease transmission. Because the transmissibility of influenza has been shown to be associated with environmental conditions [15,16], actual transmission rates could vary within the same school, house, or office building, owing to local differences in the environment. In the future, more elaborate studies should collect local environmental variables such as room ventilation rates to better quantify influenza transmission potential in confined settings [17].

In summary, Smieszek and Salathé [11] have introduced a promising and practical method to identify individuals with high infection potential who can be targeted for outbreak detection and control. Future studies should employ consistent methodological approaches to measure contact networks in different settings, in parallel with careful disease monitoring. Technological advances in contact-network sensing devices and pathogen identification methods (for example, multiplex PCR), combined with innovative approaches for disease surveillance (for example, web-based and smart-phone technologies [18]), have huge potential to increase our understanding of infectious disease transmission and to suggest novel ways of detecting and controlling outbreaks.

## Competing interests

The authors declare that they have no competing interests.

## Authors' contributions

Both authors contributed to the writing and editing of this commentary. Both authors read and approved the final manuscript.

## Authors' information

GC is an associate professor in the School of Human Evolution and Social Change at Arizona State University and a research fellow at the Fogarty International Center, US National Institutes of Health. Research interests include mathematical and statistical modeling of infectious disease transmission and control interventions, with a focus on seasonal and pandemic influenza and the quantitative characterization of past influenza pandemics.

CV is a senior research scientist at the Fogarty International Center, US National Institutes of Health, focusing on the transmission dynamics and health burden of acute viral infections, at the interface between mathematical modeling, epidemiology, evolutionary genetics, and public health.

## Pre-publication history

The pre-publication history for this paper can be accessed here:

http://www.biomedcentral.com/1741-7015/11/36/prepub
